# Immediate postpartum modern family planning utilization and associated factors among postpartum women in Gurage Zone, Southern Ethiopia 2022: community-based cross-sectional study

**DOI:** 10.3389/fgwh.2025.1355620

**Published:** 2025-04-04

**Authors:** Tolesa Gemeda Gudeta, Ayana Benti Terefe, Girma Teferi Mengistu, Seboka Abebe Sori

**Affiliations:** ^1^ Department of Nursing, College of Medicine and Health Sciences, Wolkite University, Wolkite, Ethiopia; ^2^ Department of Midwifery, College of Medicine and Health Sciences, Wolkite University, Wolkite, Ethiopia

**Keywords:** immediate postpartum, modern family planning, associated factors, Gurage Zone, Ethiopia

## Abstract

**Background:**

The period immediately following delivery is crucial for addressing the many requirements women have in terms of reproductive health, including the need for family planning after delivery and for lowering the risks associated with closely spaced pregnancies. However, contraception during the immediate postpartum period has not received enough attention in Ethiopia. Information on the use of modern family planning in the immediate postpartum period is also sparse in this study area. Therefore, the current study aimed to assess the level of immediate postpartum modern family planning utilization and associated factors among mothers who had given birth within the past twelve months in Gurage Zone, Southwest Ethiopia.

**Methods:**

The present study was conducted from May 1 to July 1, 2022, involving 844 mothers who had given birth in the previous year in the Gurage Zone of Southern Ethiopia. A community-based cross-sectional study design was utilized, with participants selected through a multistage sampling method. Data collection was performed via face-to-face interviews using a structured questionnaire. After data entry, which was done using EpiData version 3.1, analysis was carried out using the Statistical Package for Social Science (SPSS) version 26. To investigate the relationships between dependent and independent variables, both binary and multivariable logistic regressions with 95% confidence intervals were applied. In the multivariable logistic regression analysis, variables with *P*-values below 5% were considered statistically significant.

**Result:**

Altogether, a total of 836 postpartum women participated in the current study. The overall prevalence of immediate postpartum modern family planning utilization (IPPFP) was (42.9%) with 95% CI (39.6–46.3%). Attending secondary school [AOR = 1.966(1.028–3.761)], postpartum women from wealthier families [AOR = 2.57 (95% CI: 1.44–4.58)], giving birth in health facility [AOR = 2.06 *t* (95% CI: 1.26–3.38)], reporting higher women empowerment [AOR = 4.365 (2.436–7.824)], having favorable attitude [AOR = 2.65 (95% CI: 1.86–3.78)], getting counseling during ANC [AOR = 1.93 (95% CI: 1.36–2.76)] and immediate postpartum period [AOR = 2.51 (95% CI: 1.77–3.56)] were significant associated factors of IPPFP utilization.

**Conclusion:**

This study concluded that approximately two in five postpartum women utilized immediate postpartum family planning this study area. Socio-economic and informational factors significantly influence the adoption of these methods. To enhance the uptake of immediate postpartum family planning, it is essential to improve the quality of counseling provided during antenatal care visits and at the time of delivery, thereby empowering women with the knowledge needed for informed decision-making. Additionally, efforts to change attitudes toward immediate postpartum family planning through community education and awareness campaigns are vital for increasing acceptance and utilization of these services.

## Introduction

Family planning (FP) is a vital aspect of maternal and child health, with immediate postpartum family planning (IPPFP) playing a crucial role in enhancing the health and well-being of mothers and their children (1). The immediate postpartum period, defined as the first 48 h after childbirth, is a key time for initiating family planning methods, as women are particularly open to receiving counseling and starting contraception during this phase (2, 3). Adopting family planning during this time can significantly lower the risks of unintended pregnancies, maternal morbidity, and mortality. It also aids in improving birth spacing, which is essential for the health of both mothers and children (4, 5). Despite the evidence highlighting the advantages of postpartum family planning, over 120 million women worldwide require pregnancy prevention but are not using any form of family planning (6–8). In resource-limited settings, 222 million women did not have access to modern contraceptive methods within the first year after giving birth (9).

Unplanned pregnancies remain a major public health concern, especially in the year following a previous delivery (10). Such pregnancies can lead to abortion and, if they occur shortly after childbirth, may result in preterm labor, restricted fetal development, and stillbirth (11). It is also associated with increased stress, lower quality of life, delays in starting prenatal care, and higher maternal mortality rates (12, 13). Preventing unplanned pregnancies is crucial for meeting global development goals, including the Sustainable Development Goals. The World Health Organization (WHO) emphasizes that family planning should be integral to maternal and child health services (14). Moreover, utilizing effective contraception during the immediate postpartum period has proven to be a successful approach to mitigate some adverse outcomes (15). According to the WHO's 2015 Medical Eligibility Criteria for contraceptive use, women in the immediate postpartum phase have multiple family planning options, including progesterone-only pills (category 2), injectable (only for non-breastfeeding women; category 1), implants (category 2), postpartum intrauterine contraceptive devices (PPIUCD; category 1), and permanent methods like bilateral tubal ligation (category: accept) (16).

Worldwide, the decline in the number of women and girls dying annually from pregnancy complications and childbirth, from 415,000 in 2,000–295,000 in 2017, reflects a significant improvement (17). Still, approximately every two minutes, one woman dies due to complications related to pregnancy and childbirth. Most of these deaths and complications—68 percent—occur in Sub-Saharan Africa, where the maternal mortality rate stands at 533 deaths per 100,000 live births (17). In 2016, Ethiopia's maternal mortality rate was 412 deaths per 100,000 live births. This figure is slightly lower than the average for Sub-Saharan Africa, but it remains considerably higher than the global average. As an essential life-saving initiative, the Ethiopian government sought to increase the contraceptive usage rate among married women of reproductive age to 50% and reduce the unmet need for contraception to 10% as part of the second Health Sector Transformation Plan (HSTP-II) (18). However, among postpartum women who did not plan to have children soon, only 23.7% utilized modern family planning (19), and 22% of their children were born less than two years after their siblings (20). The 2019 Ethiopia Mini Demographic and Health Survey revealed that only 41% of currently married women utilize modern family planning methods (21). Moreover, immediate postpartum family planning services remain underutilized in Ethiopia, with only 9% of postpartum women using it (22).

To enhance the use of immediate postpartum family planning, the Ethiopian government and its development partners have implemented strategies such as integrating family planning services with maternal and child health services, training healthcare workers, and ensuring the availability of necessary supplies (23). However, there remains a significant gap in the utilization of immediate postpartum family planning (IPPFP) (22). This gap is especially noticeable in rural and underserved regions, where limited access to healthcare services, lack of awareness, and cultural attitudes toward contraception can obstruct the adoption of modern family planning methods (24–28). While some studies have explored IPPFP utilization in Ethiopia, they have largely overlooked the relationship between women's empowerment, wealth status, and IPPFP utilization (29, 30). Research focusing on IPPFP utilization and its influencing factors in the Gurage Zone is limited, highlighting the need for comprehensive studies to identify and understand these determinants. Therefore, the present study aimed to assess utilization of modern family planning in the immediate postpartum period and its influencing factors among postpartum women in Gurage Zone, Southwest Ethiopia. Understanding these factors is vital for developing tailored interventions that address the specific needs of postpartum women in the Gurage Zone.

## Methods and materials

### Study area and period

The present study was carried out in the Gurage Zone of the Southern Nations, Nationalities, and Peoples' Region (SNNPR) in Ethiopia. Gurage Zone is situated 153 kilometers southwest of Addis Ababa, the capital city of Ethiopia, and comprises two town administrations and 13 Woredas. According to the 2007 census by the Central Statistical Agency of Ethiopia, the total population of Gurage Zone is 1,280,483 (31). The area is served by 72 health centers and seven hospitals, which include two general zonal hospitals and five primary hospitals. The study was conducted from May 1–July 1, 2022.

### Study design

This study employed a community-based cross-sectional design to assess the uptake of immediate postpartum modern family planning and associated factors among mothers who had given birth in the past twelve months.

### Populations

All mothers who gave birth in the Gurage zone in the past twelve months (from May 1, 2021–April, 30, 2022) before the study period constituted the source population in the current study. The study population consisted of all selected mothers who gave birth in the selected Kebele in the last 12 months.

### Eligibility criteria

This study included all women who gave birth during the last 12 months and resided in the study area for at least six months and excluded those women who were unable to communicate with the interviewer, were seriously ill, and involuntary to participate.

### Sample size determination and sampling procedure

We used Epi Info version 7 StatCalc function to determine sample size by considering the following factors: a confidence interval of 95% (*Z* = 1.96), a margin of error of 5% (*d* = 0.05), and a proportion of immediate postpartum long-acting family planning method (53.2%) taken from the study done at Jimma university medical center, western Ethiopia (3) to determine the adequate sample size in the present study. Based on the above information sample size became 383, and adding 10% non-response gave us 422. However, because of the design effect, the final sample size for this study was 844. A design effect of 2 was applied in the sample size calculation.

We implemented a multistage sampling technique in the current study. Altogether, the Gurage zone has 13 woredas and two town administrations. We took six woredas and one town administration randomly by lottery method. We took four and two kebele from each randomly selected woredas and town administration, respectively. We conducted a preliminary assessment to identify eligible women in the selected Kebele with the help of health extension workers (HEW). Then, we allocated the final sample size proportionally for each kebele depending on the number of postpartum women they had. Finally, a simple random sampling technique was used to select 844 postpartum women for this study.

### Variables of the study

Immediate postpartum modern family planning utilization was a dependent variable, socio-demographic factors: age, occupation, religion, education, marital status, Obstetric related factors: gravid, parity, birth to the birth interval, the current mode of delivery, discussion with a partner to use immediate postpartum family planning, Health service-related factors: ANC visit, number of ANC visits**,** location of delivery, FP counseling during delivery and ANC, Availing FP method, and prior FP use, knowledge, attitude, women empowerment status, and household wealth status were independent variables in this study.

### Operational definitions and definition of terms

• IPPFP utilization: In this study, IPPFP utilization was defined as a postpartum woman using any modern postpartum family planning methods [such as progesterone-only pills, intrauterine contraceptive devices, injectable, dual methods, sterilization (permanent family planning method), or implants] within 48 h of delivery, prior to being discharged from the health facility (32, 33).
• Knowledge of IPPFP- In the present study, knowledge of IPPFP was assessed using 10 questions, requiring participants to respond with either “yes” or “no.” Each correct answer was assigned a value of 1, while incorrect answers received a value of 0. Scores ranged from 0–10, with the mean score used as the cut-off point. Participants were categorized as having good knowledge if their score was equal to or greater than the mean, and as having poor knowledge if their score was below the mean.
• Attitude towards IPPFP- The attitude toward IPPFP was assessed using 10 attitudinal questions. Participants rated their agreement with statements about FP on a 5-point Likert scale ranging from 1 (strongly disagree) to 5 (strongly agree). The total possible score ranged from 10–60, with the mean score serving as the cut-off point. A favorable attitude was defined as a score equal to or above the mean, while an unfavorable attitude was indicated by a score below the mean on the 10 attitudinal questions.

### Data collection tools and procedure

In the present study, we used pretested and structured instruments for data collection to achieve the study objectives, adapted the tools from the previous research (29, 34), and organized the tools into the following parts: Socio-demographics factors, reproductive histories-related factors, health service-related factors, women empowerment status, household wealth index, knowledge, and attitude-related factors (Supplementary File S2).

To assess women's empowerment status in the current study, we adapted an instrument from EDHS (35) and another relevant research (36). Women's participation in household decision-making, community group membership, access to money, home or land ownership, and educational attainment were all questions that study subjects answered to measure women's empowerment. After adding up the results, the women's empowerment index was divided into three categories: low, moderate, and high. But to make a dichotomous variable, the middle and low classes were combined during the analysis.

In the present study, the households wealth index measuring instrument was developed by Principal Component Analysis (PCA), and it was adopted from the Ethiopian Demographic Health Survey (DHS) (35) and another related study (37). More than twenty household-related variables, including fuel used for cooking, presence of a separate kitchen, land occupancy, wall, roof, and floor material, and other household properties, were included to assess the wealth indexes. We coded all these variables between 0 and 1, and then after variables were entered and analyzed using PCA, those variables having a communality value of greater than 0.5 were used to produce factor scores in this study. Finally, factor scores were summed, and for the analysis, the household's wealth index score was categorized into three categories (poor, medium, and rich).

In the current study, the face validity of the questionnaires was checked by experts with several backgrounds (An assistant professor of maternity and reproductive health nursing, an epidemiologist, and a gynecologist). Before the actual data collection, the questionnaire was translated into Amharic and re-translated to English to check the consistency of the translation. Seven Diploma and three MSC maternity health nurses who have experience in data collection and are fluent in English and local languages (Amharic and Guragigna) were recruited as data collectors and supervisors, respectively.

### Data quality management

We provided training (one day theoretical and one day practical) for data collectors and the supervisors regarding the purpose of the study, the benefit of the study, the data collection tools, and procedures, how to approach respondents, and how to maintain confidentiality before the start of data collection to maintain data quality. Supervisors and the principal investigators verified the completeness and consistency of filled questionnaires at the end of each day of data collection. In the present study, the instrument used for data collection was translated into Amharic and re-translated to English to check its consistency, pretested on 5% of the sample size to determine whether the questions were understandable and the language was appropriate before conducting the main study, following the pretest some adjustments were made, including logical ordering and rewriting of difficult-to-understand items, and the time required for data collection estimated. Each questioner was coded with unique identification and entered into Epi Data because Epi Data has a controlling mechanism for error detection. Double data entry was performed by two separate data clerks to check the consistency of data entry. A file containing the data was kept in a safe location where only the principal investigator could access it, and missing values and outliers were examined using simple frequencies and cross-tabulation in the current study.

### Data processing and analysis

The principal investigator and supervisors ensured the completeness and consistency of the data, marking the corresponding code numbers on the margins of the questionnaires. For this study, data entry was performed using EpiData version 3, and data analysis was conducted with Statistical Package for Social Science (SPSS) version 26. Descriptive statistics, including frequencies and proportions, were calculated using SPSS's descriptive statistics function. To examine the relationship between immediate postpartum modern family planning use and the independent variables, inferential statistics, such as binary logistic regression with 95% confidence intervals (CI), were applied. In the binary logistic regression model, variables with *P*-values < 0.25 were included in multivariable logistic regression. A multivariable logistic regression analysis with a backward stepwise variable selection method was conducted to adjust for confounding factors and evaluate the relative influence of independent variables on the overall use of modern family planning immediately after childbirth. In this study, we considered factors associated with IPPFP utilization using a *P*-value of <0.05 and a 95% confidence interval in the multivariable logistic regression model. None of the independent variables had a variance inflation factor (VIF) greater than 10 (all VIFs were below 3.107), and the model showed a good fit (*P*-value = 0.127) according to the Multicollinearity test sand the Hosmer and Lemeshow assumption tests.

### Ethical considerations

Before starting data collection, ethical clearance was obtained from the institutional review board of the College of Health Science and Medicine, Wolkite University, and an official letter was sent to the Gurage Zone health department to obtain permission. Data collection started after a cooperation letter was written to all districts where the study was conducted. The purpose and significance of the study were explained to the study participants, and they understood the study's objective and signed written informed consent. Before filling out the questionnaire, participants were asked for their voluntary participation, and if they did not want to continue, they were told to withdraw at any time without giving any reason, and all interviews were conducted in a private setting to ensure participant confidentiality.

## Results

### Sociodemographic characteristics of study participants

Eight hundred thirty-six (836) of the 844 invited immediate postpartum women completed the interview making a response rate of 99.05%. The current study's findings showed that the mean age of the participants was 27.0 years (SD: 5.02), and 306 (36.6%) of them fell into the 25- to 29-year-old range. Regarding the study subjects religion, marital status, and educational status, the current study results indicate that the majority of the study participants were Islam (92.5%), married (97.1%), and had completed the primary (1–8) level. Regarding women's empowerment and household wealth status, the current study demonstrated that less than three-fourths of study subjects were low/moderately empowered, and more than half of the study participants had medium wealth status (Table 1).

**Table 1:** Sociodemographic characteristics of postpartum women in the Gurage Zone, Ethiopia, 2022 (*n* = 836).

Variables	Category	Frequency	Percent (%)
Age in years	<=19	58	6.9
	20–24	219	26.2
	25–29	306	36.6
	30–34	164	19.6
	>=35	89	10.6
Marital status	Married	812	97.1
	Single	17	2.0
	Divorced/Separated	7	.8
Religion	Islam	773	92.5
	Orthodox	33	3.9
	Protestant	18	2.2
	Catholic	12	1.5
Educational status	Illiterate	99	11.8
	Primary	330	39.5
	Secondary	257	30.7
	Collage and above	150	17.9
Occupational status	Unemployed	392	46.9
	Formal Employment	154	18.4
	Informal Employment	290	34.7
Wealth status	Poor	179	21.4
	Medium	445	53.2
	Rich	212	25.4
Women empowerment status	Low	384	45.9
	Moderate	264	31.6
	High	188	22.5

### Reproductive history and family planning-related characteristics of study participants

The results showed that three-fourths of the study participants were multigravida and multipara. The results of the current study's findings regarding the study participants' mode of birth and ANC visits revealed that more than half (61.4%) of the study subjects experienced spontaneous delivery, and almost all (97.1%) of the study subjects had a history of ANC visits during a recent pregnancy. Concerning counseling for immediate postpartum family planning, more than half of the study participants received counseling for family planning during ANC visits and delivery (the immediate postpartum period). This study also revealed that Depo-Provera and implants were used by around 50.1% and 36.2%, respectively. Regarding the knowledge and attitude of study subjects towards immediate postpartum family planning, the findings of the present study revealed that more than half of the study subjects (54.2%) and 53.5% had good knowledge and positive attitude towards immediate postpartum family planning, respectively (Table 2).

**Table 2:** Reproductive history and family planning-related characteristics of postpartum women in the Gurage Zone, Ethiopia, 2022 (*n* = 836).

Variables	Category	Frequency	Percent (%)
Gravidity	Primigravida	205	24.5
	multigravida	631	75.5
Parity	Primipara	194	23.2
	Multipara	642	76.8
The current mode of delivery	Spontaneous delivery	513	61.4
	Vacuum/Forceps	66	7.9
	Cesarean Section C/S	257	30.7
Place of delivery	Hospital	319	38.2
	Health center	398	47.6
	Home	119	14.2
Needs partner approval to use PPFP	No	31	3.7
	Yes	805	96.3
Do you have ANC follow up?	No	27	2.9
	Yes	809	97.1
Counseled for immediate FP during ANC	No	391	47.9
	Yes	425	52.1
Counseled for immediate FP at immediate postpartum period	No	510	61.0
	Yes	326	39.0
Were you given information on the available FP option to start within 48 h after birth?	No	415	50.3
	Yes	421	49.7
If yes, which available options were you given the information	IUCD	17	4.0
	Implants	233	55.3
	Depo-Provera	149	35.4
	POP	22	5.2
Which family planning method you adopted within 48 h after you gave recent birth	IUCD	23	6.4
	Implants	130	36.2
	Depo-Provera	180	50.1
	POP	26	7.2
Knowledge of the participants	Poor knowledge	383	45.8
	Good knowledge	453	54.2
Attitudes of the participants	Non-favorable	389	46.5
	Favorable	447	53.5

### Utilization of IPPFP level among study participants

Overall, 359 (42.9%) of the study's participants used IPPFPs, with a 95% confidence interval (39.6%–46.3%). Depo-Provera 180 (50.1%) and Implants 130 (36.2%) were the two contraceptive methods most frequently adopted in the immediate postpartum period.

### Factors associated with IPPFP utilization

Binary and multivariate logistic regression analyzes were used in the present study to identify factors associated with IPPFP utilization. Each independent variable was separately entered into a binary logistic regression model with 95% CI. The result showed that age, educational status, occupational status, women empowerment status, wealth index, parity, having ANC visits, need partner approval to use IPPFP, attitude towards IPPFP utilization, knowledge towards IPPFP utilization, place of delivery, counseled about FP during ANC visit and delivery was candidate variable for multivariable logistic regression analysis at *p*-value < 0.25 (Table 3).

**Table 3:** Binary logistic regression results showing factors associated with adoption of immediate postpartum modern family planning method in Gurage Zone, Ethiopia, 2022 (*n* = 836).

Variables	Use modern IPPFP		*p*-value	COR (95% CI)
	No (%)	Yes (%)		
Age in years				
<=19	37 (63.8%)	21 (36.2%)		1
20–24	137 (62.6%)	82 (37.4%)	.863	1.055 (.578, 1.924)
25–29	168 (54.9%)	138 (45.1%)	.212	1.447 (.810, 2.587)
30–34	92 (56.1%)	72 (43.9%)	.308	1.379 (.743, 2.558)
>=35	43 (48.3%)	46 (51.7%)	.067	1.885 (.957, 3.713)
Occupational status				
Unemployed	251 (64.0%)	141 (36.0%)		1
Formal Employment	75 (48.7%)	79 (51.3%)	.01	1.875 (1.286, 2.735)
Informal Employment	151 (52.1%)	139 (47.9%)	.002	1.639 (1.203, 2.232)
Needs partner approval to use PPFP				
No	26 (83.9%)	5 (16.1%)		1
Yes	451 (56.0%)	354 (44.0%)	0.04	4.082 (1.55, 10.74)
Parity				
Primipara	78 (40.2%)	116 (59.8%)		
Multipara	399 (62.1%)	243 (37.9%)	0.02	2.44 (1.76, 3.39)
Place of delivery				
Hospital/health center	393 (54.8%)	324 (45.2%)	0.001	1.98 (1.30, 3.01)
Home	84 (70.6%)	35 (29.4%)		1
Wealth status				
Poor	126 (70.4%)	53 (29.6%)		1
Medium	258 (58.0%)	187 (42.0%)	0.004	1.723 (1.188, 2.500)
Rich	93 (43.9%)	119 (56.1%)	<0.001	3.042 (1.998, 4.631)
Educational status				
Illiterate	75 (75.8%)	24 (24.2%)		1
Primary (1–8)	196 (59.4%)	134 (40.6%)	0.003	2.136 (1.284, 3.556)
Secondary (9–12)	129 (50.2%)	128 (49.8%)	<0.001	3.101 (1.842, 5.219)
Collage and above	77 (51.3%)	73 (48.7%)	<0.001	2.963 (1.692, 5.188)
ANC visits				
No	18 (66.7%)	9 (33.3%)		
Yes	459 (56.7%)	350 (43.3%)	0.031	0.66 (.291, 1.48)
Women empowerment				
Low	269 (70.1%)	115 (29.9%)		1
Moderate	129 (48.9%)	135 (51.1%)	<0.001	2.45 (1.77, 3.39)
High	79 (42.0%)	109 (58.0%)	<0.01	3.227 (2.25, 4.64)
Knowledge about IPPFP				
Poor knowledge	271 (56.8%)	206 (43.2%)		
Good knowledge	112 (31.2%)	247 (68.8%)	0.17	2.901 (2.176, 3.868)
Attitude about IPPFP				
Non Favorable	277 (71.2%)	112 (28.8%)		1
Favorable	200 (44.7%)	247 (55.3%)	0.001	3.054 (2.290, 4.074)
Counseled about FP at immediate postpartum period				
No	353 (69.2%)	157 (30.8%)		1
Yes	124 (38.0%)	202 (62.0%)	0.014	3.663 (2.735, 4.905)
Counseled about FP during ANC visits				
No	259(66.2%)	132(33.8%)		1
Yes	204(48.0%)	221(52.0%)	0.01	2.126(1.602, 2.821)

Key: 1 = Reference category, COR, crude odds ratio.

Altogether, thirteen variables were entered into the multivariable logistic regression analysis to identify the final factors associated with IPPFP utilization. Backward logistic regression was employed to select variables in the final model. The results showed that women's empowerment level, wealth index, attitude towards IPPFP use, place of delivery, advice about IPPFP during ANC, and immediate delivery (childbirth) were significantly associated with IPPFP use.

In the present study, mothers who attended secondary school (9–12) were nearly two times more [95% CI: 1.03–3.76] likely to utilize immediate postpartum family planning compared with illiterate women. Postpartum women from wealthier families were 2.57 times more [95% CI: 1.44–4.58)] more likely to use immediate postpartum family planning than women from poorer families. Postpartum women who had favorable attitude towards immediate postpartum family planning were 2.65 times more [95% CI: 1.86–3.78] likely to utilize immediate postpartum family planning than women who had unfavorable attitude. Women who have been counseled about FP during immediate postpartum period were 2.51 times more [95% CI: 1.77–3.56)] and during ANC visits were 1.93 times more [95% CI: 1.36–2.76)] likely to utilize immediate postpartum modern family planning method than those who have not been counseled about family planning. Mothers who had given birth in health facility were 2.06 times more likely [95% CI: 1.26–3.38] to utilize immediate postpartum family planning than those who gave birth at home. Regarding women's empowerment, the odds of using modern family planning immediately after childbirth were 4.365 times [95% CI: 2.44–7.82] higher for women with high levels of women's empowerment than their counterparts (Table 4).

**Table 4:** Multivariable logistic regression results showing factors associated with adoption of immediate postpartum modern family planning method in Gurage Zone, Ethiopia, 2022 (*n* = 836).

Characteristics	Use modern IPPFP		COR (95% CI)	AOR (95% CI)
	No (%)	Yes (%)		
Wealth status				
Poor	126 (70.4%)	53 (29.6%)	1	1
Medium	258 (58.0%)	187 (42.0%)	1.72 (1.19, 2.50)*	1.44 (0.86, 2.39)
Rich	93 (43.9%)	119 (56.1%)	3.042 (1.99, 4.63)**	2.570 (1.44, 4.58)*
Educational status				
Illiterate	75 (75.8%)	24 (24.2%)	1	1
Primary (1–8)	196 (59.4%)	134 (40.6%)	2.136 (1.28, 3.56)*	1.29 (.705, 2.36)
Secondary (9–12)	129 (50.2%)	128 (49.8%)	3.10 (1.84, 5.22)**	1.97 (1.03, 3.76)*
Collage and above	77 (51.3%)	73 (48.7%)	2.96 (1.69, 5.19)**	.491 (.22, 1.08)
Women empowerment				
Low/moderate	390 (64.0%)	219 (36.0%)	1	1
High	87 (38.3%)	140 (61.7%)	2.87 (2.09, 3.93)**	4.37 (2.44, 7.82)**
Attitude toward IPPFP				
Non Favorable	277 (71.2%)	112 (28.8%)	1	1
Favorable	200 (44.7%)	247 (55.3%)	3.05 (2.29, 4.07)**	2.65 (1.86, 3.78)**
Place of delivery				
Home	84 (70.6%)	35 (29.4%)	1	1
Health facility	393 (54.8%)	324 (45.2%)	1.98 (1.30, 3.01)	2.06 (1.26, 3.38)*
Counseled at (immediate) delivery				
No	353 (69.2%)	157 (30.8%)	1	1
Yes	124 (38.0%)	202 (62.0%)	3.66 (2.74, 4.91)**	2.51 (1.77, 3.56)**
Counseled about IPPFP during ANC visits				
No	259 (66.2%)	132 (33.8%)	1	1
Yes	204 (48.0%)	221 (52.0%)	2.13 (1.60, 2.82)**	1.93 (1.36, 2.76)**

1 = Reference category, COR, crude odds ratio; AOR, adjusted odds ratio. *Statistically significant at *p* < 0.05.

** Statistically significant at *p* < 0.001.

## Discussion

The objective of the current study was to determine the prevalence of immediate postpartum modern family planning utilization and its associated factors among postpartum women in Gurage Zone. The results of this study indicated that the prevalence of IPPFP utilization was 359 (42.9%) with a 95% CI (39.6%–46.3%). It also revealed that IPPFP utilization is significantly associated with women's empowerment status, wealth index, place of delivery, attitude towards IPPFP utilization, and counseling about IPPFP during ANC and delivery.

The study revealed that 42.9% of postpartum women utilized modern contraceptives right after the birth of their recent child. This finding is supported by a study conducted in Addis Ababa, Ethiopia (45.5%) (38), as well as research carried out in four regions of Ethiopia (Amhara, Tigray, Oromia, and SNNP), which showed a 39% result (39). The level of immediate postpartum family planning usage we observed is higher than the rates reported in North Shoa, Ethiopia (21.3%) (30), in Konta Special Woreda, Southern Ethiopia (35.2%) (40), Saint Paul Millennium Medical College in Addis Ababa (12.7%) (29), Sidama, Ethiopia (21.6%) (41), Rwanda (28.1%) (42), and western Kenya (35.6%) (34). The higher immediate postpartum modern family planning utilization observed in this study, compared to research conducted in the aforementioned countries, may be due to variations in socio-cultural factors, study settings, and differences in the time periods of the studies. Furthermore, 97.1% of the participants in this study had attended antenatal care (ANC), which may have increased their likelihood of using immediate postpartum contraception.

However, this result is lower than the rates reported in Thailand (73.7%) (43), Jimma town in Western Ethiopia (53.2%) (3), Kenya (50%) (44), South Africa (93.2%) (45), and the United States (49%) (46). The inconsistent findings could be attributed to the presence of contraceptive counseling aimed at promoting the use of contraception right after childbirth. For example, a randomized controlled trial in Thailand examined the effects of immediate postpartum contraceptive counseling on adolescent utilization of long-acting reversible contraceptives. Additionally, the variation in results might stem from differences in the study populations. For instance, a study conducted in Kenya and South Africa focused on HIV-positive women, who were strongly encouraged to use contraceptives to limit childbirth.

The current study's finding showed a statistically significant association between the uptake of IPPFP and family planning counseling. Women who have been counseled about FP during immediate postpartum period and ANC were more likely to utilize immediate postpartum modern family planning method than those who have not been counseled about family planning. This result is supported by various studies conducted in different context, including Kebribeyah town in Eastern Ethiopia, Durame town in Southern Ethiopia, in India, Thailand, West-central Africa and a systematic review and meta-analysis in Ethiopia (43, 47–52). The consistency across these studies suggests that the positive impact of FP counseling is a widespread phenomenon, transcending cultural and geographical differences. Counseling provides women with essential information about their contraceptive options, addressing misconceptions and cultural barriers that may exist. It empowers women to make informed decisions about their reproductive health, ultimately leading to higher adoption rates of contraceptive methods immediately postpartum. Moreover, the consistency of these findings across diverse settings suggests that the relationship between counseling and IPPFP use is not limited to a specific geographic or cultural context. This reinforces the need for health systems to strengthen FP counseling during ANC visits and immediate postpartum period to enhance the uptake of family planning services, thereby contributing to better maternal and child health outcomes.

In the present study mothers who had given birth in health facility were more likely to utilize immediate postpartum family planning than those who gave birth at home. This result is corroborated with another study conducted in Ethiopia (53). The possible justification for this result might be increased access to family planning services and health education and Counseling including family planning options, which increases awareness and likelihood of utilization. Another possible justification might be attributed to the Ethiopian government's recent national policies encouraging the integration of family planning services with maternity and child health (MCH) services may also be a contributing factor, since it ensures a greater uptake among individuals who give birth in a healthcare facility.

In this study, attitudes toward contraceptive use during the immediate postpartum period were linked to actual IPPFP use. Mothers with favorable attitudes toward the IPPFP were more likely to utilize it compared to those with unfavorable attitudes. This finding is consistent with research conducted in North Shoa (30), Kebribeyah town in Eastern Ethiopia (49), and Addis Ababa (29), which demonstrated that mothers with favorable attitudes toward postpartum contraceptive methods were more likely to use it. These results suggest that favorable attitudes toward IPPFP are a precursor to its utilization across different contexts. This highlights the need for strategies aimed at changing societal attitudes toward family planning, particularly postpartum methods, by involving community leaders and influencers to promote positive perceptions and encourage utilization strengthening interventions aimed at improve the utilization. It is important to interpret this finding cautiously, as women who adopted PPFP may have more favorable attitudes on contraception.

Women's empowerment status is significantly associated with the immediate postpartum family planning method adoption. The odds of using modern family planning immediately after childbirth were 4.365 times higher among immediate postpartum women who reported experiencing higher levels of women empowerment than their counterparts. Similarly, a 2016 analysis of the EDHS (35) found that the likelihood of using health services to achieve their reproductive health goals was higher among women who described high female empowerment than those who reported low female empowerment. This result is similar to the study conducted in sub-Saharan Africa (54) and Burkina Faso (55). The possible explanation could be that women who are more empowered may have greater autonomy in making healthcare decisions, including reproductive choices. Additionally, empowered women are more likely to have access to reproductive health education and information, which improves their knowledge of FP availability, advantages, and procedures.

This study discovered that women from wealthier households were more likely to use IPPFP than women from poorer households, and this result is similar to the study conducted in Rwanda (56). The possible justification might be women from wealthier households are more likely to give birth in health facilities where IPPFP services are available, while poorer women may have limited access to institutional delivery and postpartum care. This result suggests that programs should focus on increasing women's and families' economic independence to improve women's use of family planning.

## Strengths and limitations of the study

The study employed a community-based cross-sectional design with a large sample size to ensure the reliability and representativeness of the findings. However, there are some limitations to consider. The cross-sectional nature of the data restricts the ability to infer causality between the utilization of immediate postpartum family planning (IPPFP) and its associated factors. Additionally, respondents estimated the time between giving birth to their last child and the initiation of contraceptive use, which may introduce recall bias. Some important factors, such as the combined effects of family planning counseling during antenatal care, labor and delivery, and the postpartum period, were not addressed in this study and should be explored in future research. Furthermore, there may be response bias as a result of social desirability and the dichotomization of variables including attitude and knowledge measuring items using a Likert scale.

## Conclusion and recommendation

This study concluded that approximately two in five postpartum women adopted immediate postpartum family planning in this study area. The study further demonstrates that women's empowerment, wealth index, attitudes toward IPPFP utilization, place of delivery and counseling during antenatal care (ANC) and delivery are significantly associated with the utilization of IPPFP. Therefore, health campaigns and educational initiatives aimed to improve attitudes toward IPPFP, emphasizing its benefits for maternal and child health, improving access to healthcare facilities for delivery, strengthen family planning counseling during ANC and delivery, and strengthening efforts aimed at empowering women through education, economic opportunities, and social support systems to encourage family planning uptake, including IPPFP are recommended. Future researchers are recommended to conducted mixed study.

## Data Availability

The raw data supporting the conclusions of this article will be made available by the authors, without undue reservation.
