# Supplementary material for: Immediate postpartum modern family planning utilization and associated factors among postpartum women in Gurage Zone, Southern Ethiopia 2022: community-based cross-sectional study
Source: Front Glob Womens Health. 2025 Apr 4;6:1355620. doi: 10.3389/fgwh.2025.1355620 (PMC12006149; doi:10.3389/fgwh.2025.1355620)
Supplement: Supplementary file 1 [file Datasheet1.docx]

**Questionnaire in English**

Title: Immediate Postpartum Modern Family Planning Utilization and Associated Factors among Postpartum Women in Gurage Zone, Southern Ethiopia 2022: Community-Based Cross-Sectional Study

| 1 | Where is your address? | __________ | | | | | |
| --- | --- | --- | --- | --- | --- | --- | --- |
| 2 | what is your ethinicity? | [1] Siltie [2] Hadya [3] Gurage [4] Kembata [5] Halaba [6] Oromo [7] Amhara [8] Tigray [9] Others | | | | | |
|  | what is your religion? | [1] Muslim [2] Orthodox [3] Protestant [4] Catholic [5] Others | | | | | |
| 3 | What is your age in years? | __________ | | | | | |
| 4 | How many living children do you have? | __________ | | | | | |
| 5 | What is your highest level of education? (tick one) | [1] Never attended [2] Primary school [3] Secondary [4] Tertiary [5] University | | | | | |
| 6 | What is your primary occupation? | [1] Unemployed [2] Formal employment [3] Informal employmen | | | | | |
| 7 | What is your Marital Status? | [1] Married [2] Single [3] Divorced/separated | | | | | |
| 8 | How much is your household income per month? (tick one) | [1] Less than ETB 5,000 [2] ETB 5,000-10,000 [3] ETB 10,000-50,000 [4] AboveETB 50,000 | | | | | |
| 9 | Needs partner approval to use PPFP | 1. Yes 2. No | | | | | |
| **PART II. REPRODUCTIVE HISTORIES RELATED FACTORS** | | | | | | | |
| 1 | Number of pregnancy | __________ | | | | | |
| 2 | No of parity | __________ | | | | | |
| 3 | Current mode of delivery | 1. SVD 2. Vacuum/ Forceps delivery 3. C/S | | | | | |
| **PART II. HEALTH SERVICE RELATED FACTORS** | | | | | | | |
| 1 | ANC visit | 1.Yes 2. No | | | | | |
| 2 | Number of ANC visit | ­­­­__________ | | | | | |
| 3 | Counselled for immediate FP during Antenatal care | 1. Yes 2, No | | | | | |
| 4 | Place of Delivery for the recent child | 1. Health centre 2. At home 3. Hospital | | | | | |
| 5 | Were you given information on the available FP option to start within 48hours after birth? | 1. Yes 2. No | | | | | |
| 6 | If yes, which available options were you given the information | [1] IUCD [2] Implants [3] Depo-Provera [4] Progesterone only pill (POP) [5] Combined Oral Contraceptives (COC) [6]Female condoms | | | | | |
| 7 | Which family planning method you adopted within 48hours after you gave recent birth | [1] IUCD [2] Implants [3] Depo-Provera [4] Progesterone only pill (POP) [5] Combined Oral Contraceptives (COC) [6]Female condoms | | | | | |
| **PART IV: KNOWLEDGE RELARED FACTOR** | | | | | | | |
| 1 | Ever heard of IPPFP | 0. No 1. Yes | | | | | |
| 2 | know IPPFP used to prevent unwanted pregnancy | 0. No 1. Yes | | | | | |
| 3 | know IPPFP used to space pregnancy | 0. No 1. Yes | | | | | |
| 4 | know IPPFP used to limit pregnancy | 0. No 1. Yes | | | | | |
| 5 | Know condoms used to prevent pregnancy | 0. No 1. Yes | | | | | |
| 6 | Know side effects of contraceptives | 0. No 1. Yes | | | | | |
| 7 | Know exclusive breastfeeding used as family planning | 0. No 1. Yes | | | | | |
| 8 | Know that fertility resumed after stopping contraceptive | 0. No 1. Yes | | | | | |
| 9 | Know FP is given free of charge in Ethiopia | 0. No 1. Yes | | | | | |
| 10 | IPPFP can be used by HIV positive patients doing well on treatment | 0. No 1. Yes | | | | | |
| **PART V: ATTITUDE RELATE FACTORS** | | | | | | | |
|  |  | | **SD** | **D** | **N** | **A** | **SA** |
| 1 | Adopt contraceptive in the future | |  |  |  |  |  |
| 2 | Encourage your friend to adopt PPFP | |  |  |  |  |  |
| 3 | Discussing PPFP use with partner is good | |  |  |  |  |  |
| 4 | Using postpartum contraceptive is shame | |  |  |  |  |  |
| 5 | PPFP is good for mother and child health | |  |  |  |  |  |
| 6 | Small family size makes family happy | |  |  |  |  |  |
| 7 | PPFP use good for standard of living | |  |  |  |  |  |
| 8 | Exclusive BF used to prevent pregnancy | |  |  |  |  |  |
| 9 | Unmarried women can use contraceptive | |  |  |  |  |  |
| 10 | using Contraceptive can cause infertility | |  |  |  |  |  |

| **PART VI:** **Socio-economic information** | | | | | | |  |
| --- | --- | --- | --- | --- | --- | --- | --- |
| 1 | Does your household have?  A television  A mobile telephone?  A landline telephone?  A refrigerator?  A bed?  A sofa?  A cupboard?  A computer?  A fan?  A clock  A dhiki/janto | ☐ Yes ☐ No  ☐ Yes ☐ No  ☐ Yes ☐ No  ☐ Yes ☐ No  ☐ Yes ☐ No  ☐ Yes ☐ No  ☐ Yes ☐ No  ☐ Yes ☐ No  ☐ Yes ☐ No  ☐ Yes ☐ No  ☐ Yes ☐ No | | Yes=1  No=0 | |  |  |
| 2 | What type of fuel does your household mainly use for cooking? | ☐ Electricity  ☐ LPG  ☐ Biogas  ☐ Kerosene  ☐ Wood  ☐ Animal dung  ☐ Others….(specify) | | 1  2  3  4  5  6  99 | |  |  |
| 3 | Do you have a separate room, which is used, as a kitchen? | ☐ Yes  ☐ No | | 1  0 | |  |  |
| 4 | Main material of the floor | ☐ Earth/dung  ☐ Cemented  ☐ Ceramic tiles  ☐ Finished floor/parquet  ☐ Other (specify)………… | | 1  2  3  4  99 | | Observe and write |  |
| 5 | Main material of the roof | ☐ Thatched roof  ☐ Galvanized sheet  ☐ Ceramic tiles  ☐Cement  ☐ Other (specify)………. | | 1  2  3  4  99 | | Observe and write |  |
| 6 | Main materials of the exterior walls | ☐Tin  ☐Mud and stone/brick  ☐Wood/bamboo  ☐Concrete  ☐Other (specify)………… | | 1  2  3  4  99 | | Observe and write |  |
| 7 | How many rooms in this household are used for sleeping? | ……..rooms | |  | |  |  |
| 8 | Does any member of this household own  A bicycle/ rickshaw?  A motorcycle or motor scooter?  A three wheel tempo?  A tractor?  A car or truck? | ☐ Yes ☐ No  ☐ Yes ☐ No  ☐ Yes ☐ No  ☐ Yes ☐ No  ☐ Yes ☐ No | | Yes=1  No=0 | |  |  |
| 9 | Does any member of this household own any agricultural land? | ☐ Yes  ☐ No | | 1  0 | | If 0= skip to Q.N. 11 |  |
| 10 | If yes, how many hectares of agricultural land do members of this household own? | ☐ Less than 1…………  ☐ Greater than1………. | | 1  2 | |  |  |
| 11 | Does this household own any livestock, herds, other farm animals or poultry? | ☐ Yes  ☐ No | | 1  0 | |  |  |
| 12 | How many of the following animals does this household own? | ☐ Buffalo ………………  ☐ Cow and bull ………..  ☐ Goat …………………  ☐ Chicken/duck ………..  ☐ Pig ………………….. | |  | | If 0= skip to Q.N. 13 |  |
| 13 | Does your household own any kitchen garden? | ☐ Yes  ☐ No | |  | | If 0= skip to Q14-16 |  |
| 14 | What have you grown in your kitchen garden? | ....................  …………………………. | |  | |  |  |
| 15 | Does your kitchen garden product is sufficient for your family consumption? | ☐ Yes  ☐ No | | 1  0 | |  |  |
| 16 | In the past four weeks, how often did your household bought vegetables for consumption? | ☐ Never  ☐ Rarely  ☐ Sometimes  ☐ Often | | 1  2  3  4 | |  |  |
| **PART VII: Information on women’s empowerment** | | | | | | | |
| 1 | Do you belong to any group? Please specify.  Mother’s group  Saving/ co-operative group Women’s group | | ☐Yes ☐ No  ☐ Yes ☐ No  ☐ Yes ☐ No | | Yes=1  No=0 | |  |
| 2 | Do you own this or any other house either alone or jointly with someone else? | | ☐ Alone only  ☐ Jointly only  ☐ Both alone and jointly  ☐ Does not own | | 1  2  3  4 | |  |
| 3 | Do you own any land either alone or jointly with someone else? | | ☐ Alone only  ☐ Jointly only  ☐ Both alone and jointly  ☐ Does not own | | 1  2  3  4 | |  |
| 4 | Who usually makes decisions about health care for yourself | | ☐ Participant only  ☐ Husband only  ☐ Participant and husband jointly  ☐ Other (specify)…........... | | 1  2  3  99 | |  |
| 5 | Who usually makes decisions about making major household purchases? | | ☐ Participant only  ☐ Husband only  ☐ Participant and husband jointly  ☐ Other (specify)…........... | | 1  2  3  99 | |  |
| 6 | Who usually makes decisions about visits to your family or relatives? | | ☐ Participant only  ☐ Husband only  ☐ Participant and husband jointly  ☐ Other (specify)…........... | | 1  2  3  99 | |  |
